# Integrated qPCR and Shotgun Metagenomics for Surveillance of Antimicrobial Resistance in Municipal Wastewater from Central Italy

**DOI:** 10.3390/antibiotics15090850

**Published:** 2026-08-31

**Authors:** Federica Di Timoteo, Emanuela Di Giulio, Marco Di Domenico, Barbara Secondini, Francesca Marotta, Giusy Matteucci, Katiuscia Zilli, Teresa Romualdi, Dalia Palmieri, Giuliano Garofolo, Anna Janowicz

**Affiliations:** 1 National Reference Laboratory for Campylobacter, Istituto Zooprofilattico Sperimentale dell’Abruzzo e del Molise “G. Caporale”, 64100 Teramo, Italy; e.digiulio@izs.it (E.D.G.); f.marotta@izs.it (F.M.); g.matteucci@izs.it (G.M.); k.zilli@izs.it (K.Z.); t.romualdi@izs.it (T.R.); g.garofolo@izs.it (G.G.); a.janowicz@izs.it (A.J.); 2 National Reference Centre (NRC) for Whole Genome Sequencing of Microbial Pathogens: Database and Bioinformatics Analysis (GENPAT), Istituto Zooprofilattico Sperimentale dell’Abruzzo e del Molise (IZSAM), 64100 Teramo, Italy; m.didomenico@izs.it (M.D.D.); b.secondini@izs.it (B.S.); 3 Prevention, Food Safety and Veterinary Service, Abruzzo Region Health Department, 65124 Pescara, Italy; dalia.palmieri@regione.abruzzo.it

**Keywords:** antimicrobial resistance, wastewater treatment plant, metagenome

## Abstract

**Background/Objectives:** Wastewater-based epidemiology (WBE) has emerged as a valuable One Health approach for monitoring antimicrobial resistance (AMR) at the population level. Although quantitative PCR (qPCR) and shotgun (SG) metagenomics are widely used for wastewater surveillance, studies integrating these complementary approaches remain limited. This study aimed to investigate the occurrence, seasonal dynamics, and diversity of antimicrobial resistance genes (ARGs) in municipal wastewater from Central Italy by combining targeted qPCR and SG metagenomic sequencing. **Methods:** Influent wastewater samples were collected monthly from eight municipal wastewater treatment plants in Central Italy between April 2025 and March 2026. Clinically relevant antimicrobial resistance genes were quantified by quantitative real-time PCR, while SG metagenomic sequencing was used to characterize resistome composition, resistance gene families, and ARG sequence diversity using bioinformatic pipelines. **Results:** All investigated ARGs were detected in every sample. Significant seasonal variation was observed for all investigated markers, including *qnrS*, *blaKPC*, *blaCTX-M* and *intI1*. Metagenomic analysis revealed broadly similar resistome profiles across sampling sites and time points, dominated by resistance genes to macrolide–lincosamide–streptogramin, aminoglycosides, β-lactams, and fluoroquinolones. High sequence diversity was observed within the dominant ARG families, highlighting the complementary value of SG metagenomics for comprehensive resistome characterization. **Conclusions:** The integration of targeted qPCR and SG metagenomics provided a comprehensive characterization of antimicrobial resistance in municipal wastewater. While qPCR enabled sensitive quantification of clinically relevant ARGs and revealed seasonal trends, metagenomics expanded resistome characterization by identifying dominant resistance classes, gene families, and sequence variants. These findings support the implementation of integrated molecular approaches for routine wastewater-based AMR surveillance within a One Health framework.

## 1. Introduction

Antimicrobial resistance (AMR) is recognized as one of the greatest global threats to public health, posing a major challenge to the sustainability of healthcare systems [1]. The extensive use of antibiotics in human medicine, veterinary practice, and agriculture has accelerated the emergence and dissemination of antibiotic-resistant bacteria (ARB) and antibiotic resistance genes (ARGs). These resistance determinants can spread among diverse bacterial populations through horizontal gene transfer (HGT) mechanisms. Consequently, AMR has evolved into a complex One Health issue that closely links human, animal, and environmental health [2].

Recognizing the global impact of AMR, the World Health Organization and the European Commission have emphasized the need to extend AMR surveillance beyond clinical settings to include environmental compartments, particularly urban wastewater [1,3]. Urban wastewater represents an important reservoir of antimicrobial resistance determinants and, by integrating microbial and resistome signatures originating from the population served by the sewer network, provides an aggregate picture of resistance circulating within a community [4]. Wastewater treatment plants (WWTPs), in turn, represent a critical interface between anthropogenic wastewater and receiving environments, continuously receiving microorganisms, antibiotic residues, ARGs, and other contaminants from multiple sources, including households, healthcare facilities, industrial activities, and potentially agricultural inputs.

Wastewater and WWTPs therefore play distinct but interconnected roles in the environmental dimension of AMR. While wastewater provides an integrated matrix for population-level AMR surveillance, WWTPs represent key control points influencing the subsequent fate of resistance determinants before their release into receiving environments. Although wastewater treatment substantially reduces microbial loads, ARB and ARGs may not be completely removed during treatment and can persist in treated effluents and subsequently reach aquatic environments [5,6]. Furthermore, the convergence of diverse bacterial populations, antimicrobial residues, and mobile genetic elements within WWTPs may provide conditions favoring the persistence and horizontal dissemination of resistance determinants [7,8]. Assessing these processes requires investigation across different treatment stages; conversely, monitoring untreated influent wastewater provides information on AMR circulating within the population contributing to the sewer catchment.

In this context, wastewater-based epidemiology (WBE) has emerged as a promising approach for environmental AMR surveillance. Wastewater surveillance can provide population-level information on the occurrence and temporal dynamics of antimicrobial resistance, complementing traditional clinical surveillance. Over the last decade, this approach has been increasingly applied across diverse geographical settings, demonstrating the potential of urban wastewater for monitoring AMR at the population level [9,10,11].

Molecular approaches have played a central role in the development of wastewater-based AMR surveillance. Quantitative PCR (qPCR) enables sensitive and targeted quantification of clinically relevant ARGs, whereas shotgun (SG) metagenomic sequencing provides untargeted characterization of resistome composition and diversity. Comparative studies have demonstrated that these approaches provide complementary information, with targeted methods offering high sensitivity for selected resistance determinants and metagenomics enabling broader resistome profiling [12,13].

Longitudinal investigations have shown that wastewater ARG profiles can vary over time, with several factors, including antimicrobial consumption and bacterial community composition, proposed as potential contributors to these variations [14,15]. However, temporal and seasonal patterns are not necessarily consistent among individual ARGs, wastewater matrices, or geographical settings [16]. These observations highlight the importance of longitudinal monitoring for distinguishing persistent resistome features from temporal fluctuations and for interpreting changes in ARG abundance within specific study contexts.

In Italy, several studies have applied molecular approaches to characterize ARG occurrence, persistence, and diversity in wastewater and WWTPs [17,18,19,20,21,22,23]. However, the integration of targeted qPCR and SG metagenomics within longitudinal, multi-site surveillance frameworks remains comparatively limited. Combining these approaches provides the opportunity to monitor temporal changes in selected clinically relevant resistance determinants while simultaneously characterizing the broader resistome and its diversity across wastewater catchments.

Therefore, the aim of this study was to investigate the occurrence and temporal dynamics of selected clinically relevant antimicrobial resistance markers (*blaKPC*, *blaCTX-M*, *qnrS*) and *intI1* in untreated influent wastewater collected from eight urban WWTPs in Central Italy over a one-year monitoring period using quantitative real-time PCR. In parallel, SG metagenomic sequencing was employed to characterize the wastewater resistome, with particular emphasis on the distribution and diversity of resistance genes associated with the predominant antimicrobial resistance classes. By integrating targeted and untargeted molecular approaches, this study aimed to evaluate their complementary contribution to wastewater-based AMR surveillance within a One Health framework.

## 2. Results

### 2.1. Study Workflow

A total of 96 influent wastewater samples were analysed by qPCR to determine the occurrence and temporal dynamics of four clinically relevant antimicrobial resistance markers (*blaKPC*, *blaCTX-M*, *qnrS*, and *intI1*). Subsequently, 36 samples were subjected to SG metagenomic sequencing to provide a comprehensive characterization of the wastewater resistome. Sample selection was designed to capture both temporal and spatial variability across the monitored WWTPs. To investigate temporal resistome dynamics, monthly samples collected from S5 (Abruzzo region) and S6 (Molise region) between April 2025 and March 2026 (24 samples) were analysed. In addition, to assess spatial and seasonal variability, samples collected from all eight WWTPs (S1–S8) during two monitoring campaigns (July 2025 and January 2026), corresponding to summer and winter, were analysed.

### 2.2. Quantification of 16S rRNA Gene and ARGs

The absolute abundance (gene copies/mL) and relative abundance (gene copies normalized to 16S rRNA gene copies) were determined for ARGs and *intI1* at each sampling site (Supplementary Table S1). All analysed samples tested positive for the investigated target genes. The 16S rRNA gene showed the highest concentrations overall, reflecting the total bacterial load in wastewater samples. Absolute concentrations ranged from 6.45 × 10^6^ to 3.07 × 10^9^ gene copies/mL. In comparison, ARGs were detected at lower absolute abundances but were consistently present across all sampling sites and sampling periods, with concentrations ranging between 1.00 and 7.17 × 10^6^ gene copies/mL. Among the investigated ARGs, *qnrS* was the most abundant gene overall, whereas *blaKPC* and *blaCTX-M* displayed comparable concentration levels.

### 2.3. Temporal Dynamics of AMR Genes

**Figure 1:**
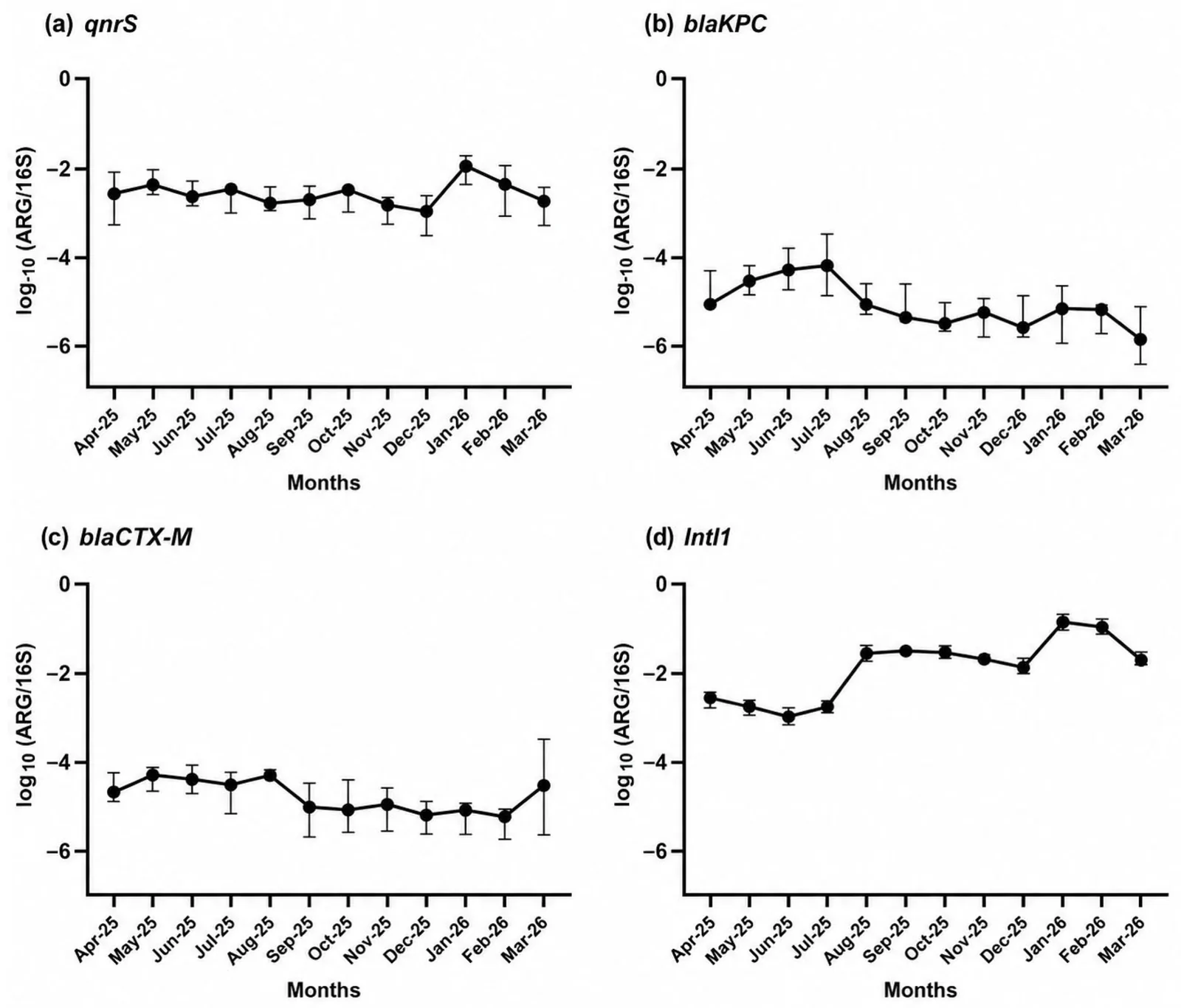
Monthly variation in the relative abundance of *qnrS* (**a**), *blaKPC* (**b**), *blaCTX-M* (**c**) and *intI1* (**d**) quantified by qPCR in wastewater samples collected from eight wastewater treatment plants between April 2025 and March 2026. Relative abundance is expressed as the log10-transformed ratio between target gene copies and 16S rRNA gene copies [log10 (ARG/16S)]. Data are presented as monthly medians (n = 8), with error bars indicating the interquartile range (IQR).

A temporal variation in the relative abundance of the investigated AMR genes was observed throughout the sampling period (Figure 1). Overall, distinct temporal patterns were identified for the different resistance markers, indicating that each marker exhibited a distinct temporal pattern. For *qnrS*, relative abundance fluctuated throughout the study period, with median log10-transformed values ranging from −2.97 to −1.96 log10 (ARG/16S). Lower median values were generally observed during late summer and autumn, whereas the highest relative abundance was recorded in January 2026, followed by a slight decrease during the subsequent months. The relative abundance of *blaKPC* and *blaCTX-M* also exhibited marked temporal variability. Median log10-transformed values ranged from −5.59 to −4.19 log10 (ARG/16S) for *blaKPC* and from −5.21 to −4.28 log10 (ARG/16S) for *blaCTX-M*. Both genes displayed higher relative abundances during spring and summer, followed by a decline from autumn onwards, with the lowest levels generally observed during winter.

Among all investigated markers, *intI1* displayed the most pronounced temporal variation. Median log10-transformed relative abundance values ranged from −2.97 to −0.85 log10 (ARG/16S). Following relatively low abundances during spring and early summer, *intI1* increased progressively from late summer onwards, remained elevated throughout autumn, and peaked during winter, reaching approximately 100-fold higher relative abundance than the lowest levels observed during the study period, before declining again towards the end of the study.

**Figure 2:**
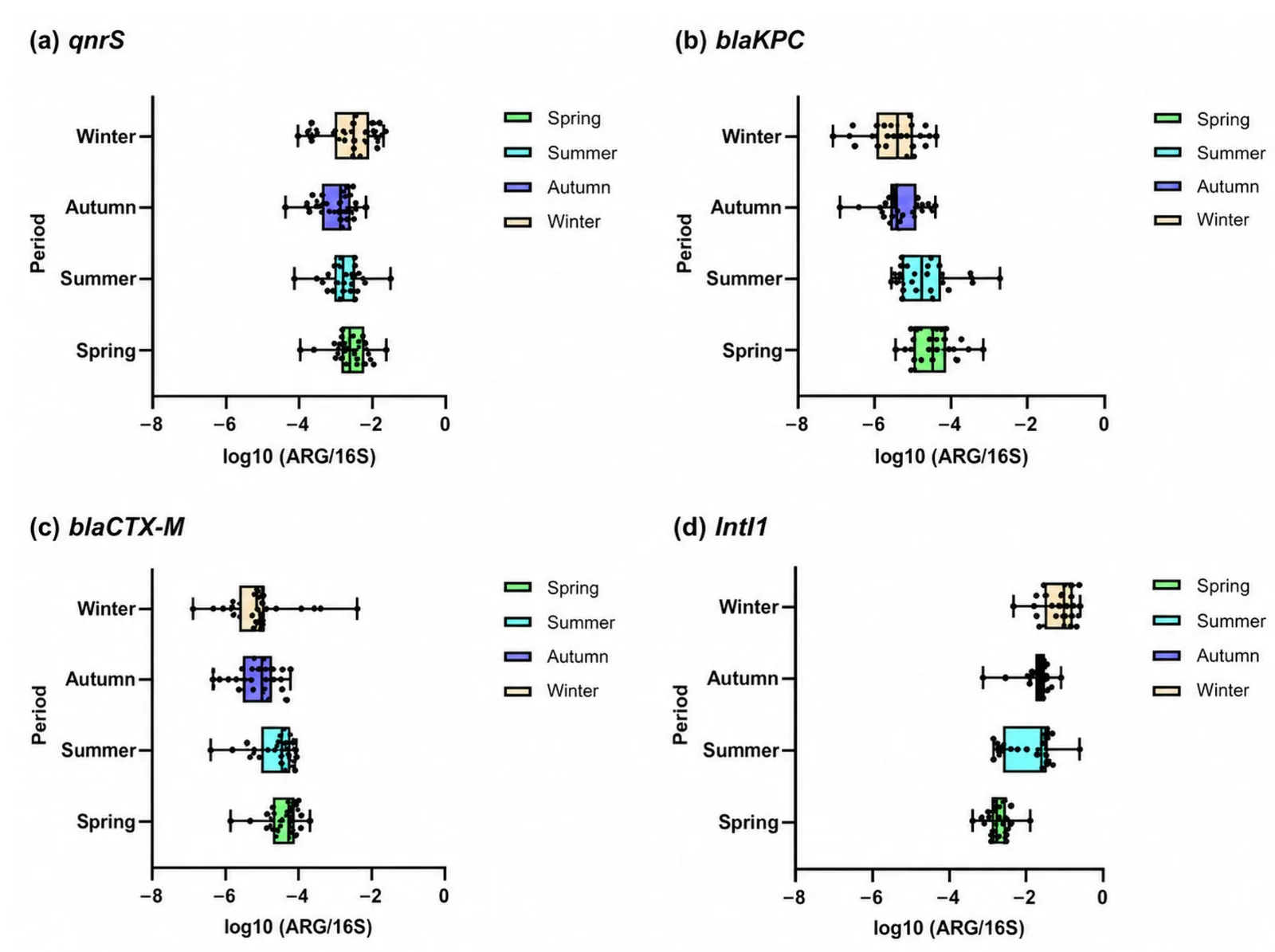
Seasonal distribution of the relative abundance of *qnrS* (**a**), *blaKPC* (**b**), *blaCTX-M* (**c**), and *intI1* (**d**) quantified by qPCR in wastewater samples collected from eight wastewater treatment plants. Relative abundance is expressed as log10 (ARG/16S). Each point represents one wastewater sample (n = 24 per season). Boxplots show the median, interquartile range (IQR), and whiskers extending to 1.5 × IQR. Seasonal differences were significant for *blaKPC*, *blaCTX-M*, *qnrS* and *intI1* (*p* < 0.05).

Aggregation of the data by season further highlighted temporal differences observed during the one-year study period (Figure 2). To account for repeated sampling of the same WWTPs, seasonal differences were assessed using the Friedman test, which revealed a significant overall seasonal effect for all investigated resistance markers: *qnrS* (*p* = 0.018), *blaKPC* (*p* = 0.002), *blaCTX-M* (*p* < 0.001), and *intI1* (*p* < 0.001).

### 2.4. Metagenomic Profiling of Antimicrobial Resistance

A total of 36 wastewater samples were sequenced and included in the resistome analysis. Sequencing generated between 12.9 and 82.0 million paired-end reads per sample (median: 45.6 million reads). ARG-associated reads accounted for 0.50–1.26% of the quality-filtered metagenomic reads, indicating that antimicrobial resistance genes constituted a relatively small but consistently detectable fraction of the wastewater metagenome (Supplementary Table S2).

**Figure 3:**
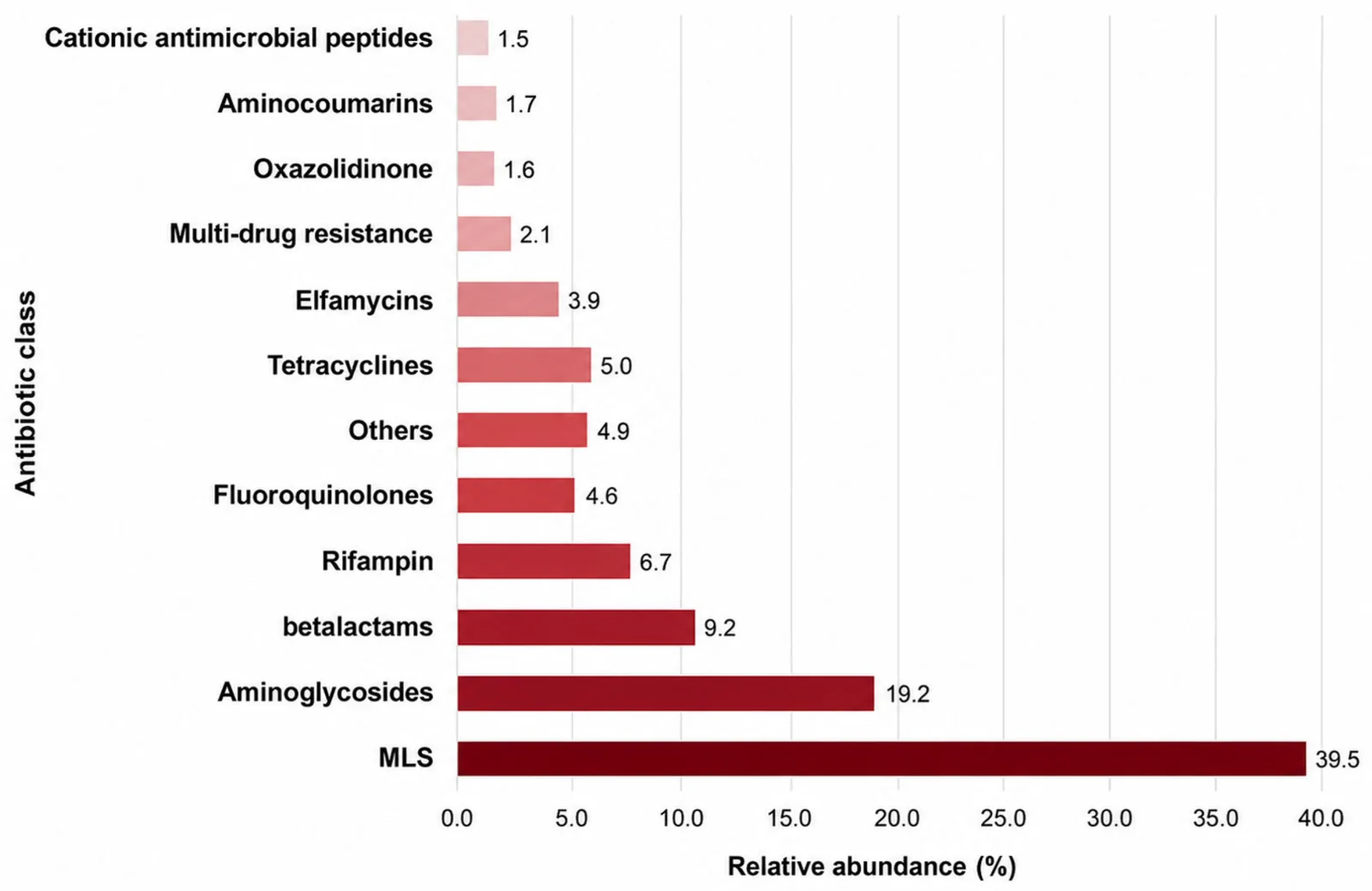
Median relative abundance of ARG classes identified by AMR++ across the 36 wastewater metagenomes.

ARG abundance was quantified using the AMR++ pipeline and grouped according to antibiotic resistance class. Overall, the resistome showed broadly similar compositions across the investigated samples, with genes conferring resistance to macrolide–lincosamide–streptogramin (MLS), aminoglycosides, β-lactams, rifamycins and fluoroquinolones accounting for the majority of the detected ARGs (Figure 3).

Comparison of the eight WWTPs sampled in July 2025 and January 2026 showed broadly similar ARG-class compositions across the investigated sites and sampling periods. MLS remained the dominant resistance class in both surveys, followed by aminoglycoside and β-lactam resistance genes. (Supplementary Figure S1).

### 2.5. Composition of Clinically Relevant Resistance Gene Families

Given the broadly similar overall resistome composition observed across the investigated samples, clinically relevant resistance classes were further characterized at the gene-family level using the AMR++ pipeline.

Overall, the greatest sequence diversity was observed among β-lactam resistance determinants, with 167 variants identified across the detected β-lactamase families, followed by aminoglycoside (45 variants), MLS (35 variants) and fluoroquinolone resistance determinants (14 variants).

MLS resistance displayed a different organization, being largely supported by macrolide phosphotransferases (mph) and 23S rRNA methyltransferases (*erm*). The *mph* family accounted for approximately 29–74% of the MLS resistome, whereas *erm* genes represented 18–45%. Macrolide esterases (*ere*) and lincosamide nucleotidyltransferases (*lnu/lin*) contributed smaller fractions, while *vat* and *vgb* genes were consistently detected at very low abundances.

Aminoglycoside resistance was characterized by the predominance of ANT(3″) and AAC(6′) genes, accounting for approximately 12–35% and 17–28% of the aminoglycoside resistome, respectively. APH(3′), AAC(3), APH(6) and ANT(6) formed a second group of consistently detected resistance families, whereas the remaining determinants individually contributed only marginally to the overall aminoglycoside resistome. Overall, the composition of aminoglycoside resistance genes varied only slightly among samples.

β-lactam resistance was dominated by OXA-like β-lactamases, which accounted for approximately 30–69% of the β-lactam resistome across all samples. GES-, FOX- and CTX-like β-lactamases represented secondary families, generally contributing less than 5%, whereas IMP, KPC, VIM and NDM remained only minor components. Although a transient decrease in the relative abundance of OXA-like genes was observed in a limited number of samples, the overall distribution of the major β-lactam resistance families was broadly similar across the investigated samples.

The fluoroquinolone resistome was driven primarily by *qepA*, which represented the predominant determinant across most samples, followed by *qnrS*, while *qnrB* and *qnrVC* contributed intermediate proportions. The remaining PMQR determinants (*qnrA*, *qnrC*, and *qnrD*) consistently represented only a small fraction of the fluoroquinolone resistome, resulting in a similar distribution of the major fluoroquinolone resistance determinants across the investigated samples.

**Figure 4:**
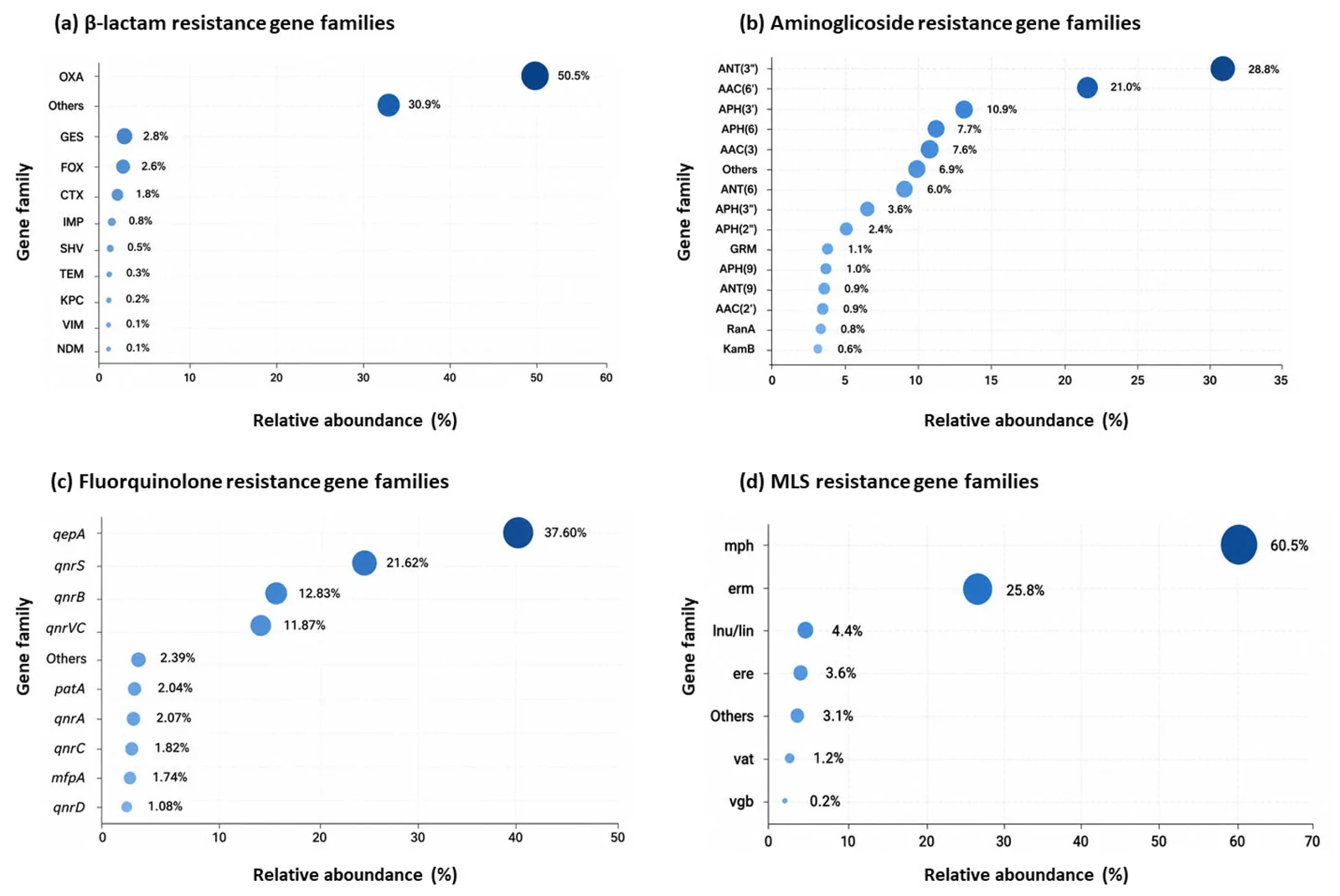
Median relative abundance of the major antimicrobial resistance gene families detected by AMR++ in municipal wastewater. Bubble plots illustrate the distribution of gene families within the (**a**) β-lactam, (**b**) aminoglycoside, (**c**) fluoroquinolone and (**d**) MLS resistance classes. Circle size and color intensity are proportional to the relative abundance of each resistance gene family.

The overall composition of the major resistance gene families is summarized in Figure 4, whereas the complete relative abundance ranges for each gene family are reported in Supplementary Tables S3–S6.

### 2.6. Diversity and Prevalence of ARG Variants

To investigate ARG sequence diversity at the variant level, metagenomic reads were additionally analyzed using the CZ ID platform for taxonomic classification. For consistency with the AMR++ analysis, resistance determinants were grouped into the same major resistance classes, with β-lactam resistance including genes conferring resistance to penams, cephalosporins, carbapenems, cephamycins, penems and monobactams, and MLS including macrolide-, lincosamide- and streptogramin-resistance genes. Overall, sequence diversity across resistance classes followed the order β-lactams (167 variants) > aminoglycosides (45 variants) > MLS (34 variants) > fluoroquinolones (14 variants).

Within the MLS class, the *erm* family displayed the greatest diversity (14 variants), followed by *lnu/lin* (9 variants), *mph* (6 variants) and *ere* (4 variants), while *mrx* was represented by a single variant. At the individual variant level, *ermB*, *ermF*, *mphA*, *mphE*, *mphG* and *mrx* were detected in all samples (100%), while *lnuH* was detected in 94.4%. Among macrolide esterase genes, *ereA2*, *ereD* and *ereA* were the most prevalent variants, occurring in 91.7%, 80.6% and 72.2% of samples, respectively.

Aminoglycoside resistance determinants were mainly represented by the *aad* group, with 21 variants, followed by AAC (9 variants), ANT (8 variants) and APH (7 variants). At the individual variant level, AAC(6′)-Ib7, APH(3″)-Ib, APH(6)-Id, ANT(3″)-IIa, *aadA6*, *aadA13* and *aadA27* were detected in all samples (100%), while *aadA5* was detected in 97.2% of samples. AAC(3)-Ia and ANT(3″)-II-AAC(6′)-IId were also highly prevalent, each occurring in 83.3% of samples.

The high sequence diversity observed for β-lactam resistance was largely attributable to OXA β-lactamases, which accounted for 109 variants. GES (20 variants), FOX (13 variants), TEM (11 variants), IMP (5 variants), SHV (5 variants) and VIM (4 variants) contributed to a lesser extent, whereas KPC, CTX-M and NDM β-lactamases were not detected. At the individual variant level, OXA-205 was the most prevalent (94.4%), followed by OXA-333 (91.7%), OXA-464 (88.9%), OXA-211 (86.1%) and OXA-20 (80.6%).

Fluoroquinolone resistance determinants were predominantly represented by plasmid-mediated quinolone resistance (PMQR) genes. The *qnrS* family showed the greatest diversity (5 variants), followed by *qnrVC* and *qnrB* (4 variants each), while *qnrD* was represented by a single variant. At the individual variant level, *qnrS2* was the most prevalent determinant (80.6%), followed by *qnrVC4* (66.7%) and *qnrS6* (52.8%). All remaining variants occurred in fewer than 25% of samples.

**Figure 5:**
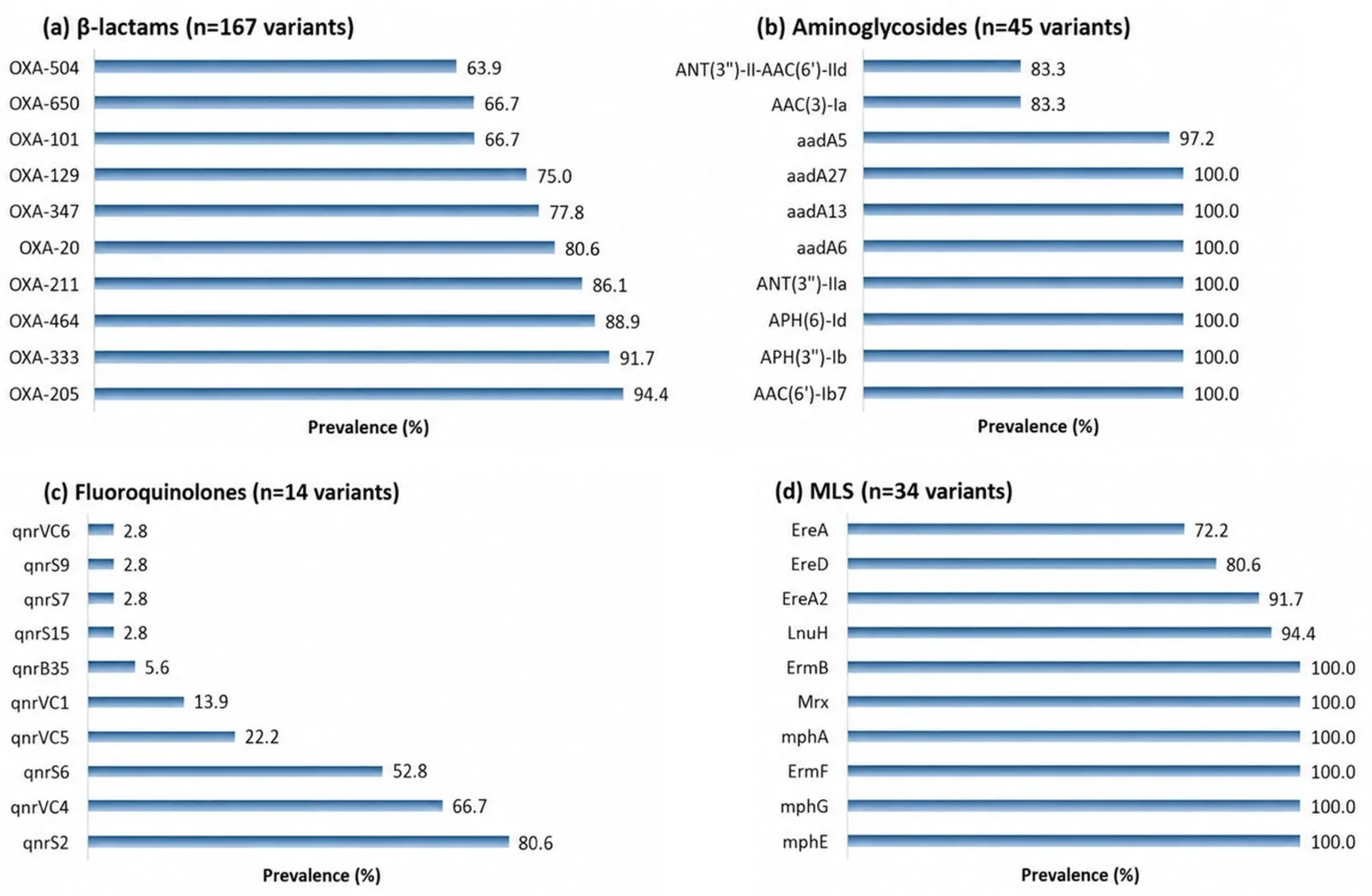
Diversity of clinically relevant antimicrobial resistance gene variants detected by CZ ID tools. Horizontal bar plots showing the number of distinct variants identified for the major gene families within (**a**) β-lactam (n = 167 variants), (**b**) aminoglycoside (n = 45 variants), (**c**) fluoroquinolone (n = 14 variants) and (**d**) (MLS) (n = 34 variants) resistance classes across all wastewater samples.

The prevalence of the principal ARG variants identified by the CZ ID antimicrobial resistance workflow is summarized in Figure 5, whereas the complete distribution of all detected ARG variants is provided in the Supplementary Tables S7–S10.

## 3. Discussion

The present study provides a year-long characterization of AMR in untreated municipal wastewater from Central Italy by integrating targeted qPCR and SG metagenomic sequencing. The results revealed the widespread occurrence of clinically relevant ARGs, distinct temporal dynamics among individual resistance markers, and broadly similar distributions of the major resistance classes across the investigated samples.

The consistent detection of *qnrS*, *blaCTX-M*, *blaKPC*, and *intI1* in all samples confirms the widespread occurrence of clinically relevant resistance determinants in untreated municipal wastewater. Similar observations have been reported in Italian wastewater surveillance studies, where *blaCTX-M*, *blaKPC*, and *intI1* were repeatedly detected during longitudinal monitoring [17,21]. Comparable abundance patterns have also been described in WWTPs serving urban and rural catchments in northern Italy, where *qnrS* and *intI1* were among the most abundant targets, whereas *blaCTX-M* and *blaKPC* generally occurred at lower relative abundances [22]. The comparatively higher abundance of *qnrS* and *intI1* observed in the present study may reflect their broader distribution within wastewater bacterial communities, whereas clinically relevant β-lactamase genes may occur within a more restricted range of bacterial hosts. Nevertheless, differences in target selection, normalization procedures, wastewater composition, and catchment characteristics should be considered when comparing ARG abundances among studies. These findings are also consistent with national-scale wastewater surveillance studies reporting clinically relevant ARGs across multiple sampling locations [13,24].

Despite their ubiquitous occurrence, the investigated targets exhibited different temporal dynamics during the one-year monitoring period. Analysis of data grouped by season and accounting for repeated measurements revealed significant differences among seasons for all four markers, although the magnitude and direction of these variations differed among ARGs. While *qnrS* showed relatively moderate fluctuations, *blaCTX-M* and *blaKPC* tended to exhibit higher relative abundances during warmer periods, whereas intI1 progressively increased towards winter. Temporal variations in wastewater ARG abundance have also been reported in longitudinal studies, although their direction and magnitude are not consistent. Caucci et al. (2016) observed higher relative ARG abundances during autumn and winter over a two-year monitoring period, coinciding with increased outpatient antibiotic prescriptions [14]. Guruge et al. (2025) similarly reported seasonal variation in the resistome of activated sludge, with enrichment of several high-risk ARGs during the cold season and a contribution of bacterial community composition to these patterns [15]. Environmental and hydrological factors, including precipitation, have also been proposed as potential contributors to temporal ARG variation [25]. Together, these observations suggest that temporal dynamics may reflect multiple interacting anthropogenic, microbiological, and environmental factors rather than season alone. However, antibiotic consumption, bacterial community composition, and environmental drivers were not investigated in the present study and therefore represent possible explanations rather than mechanisms directly supported by our data. Consistent with the heterogeneous seasonal patterns reported across wastewater studies [16], and considering that our monitoring covered a single annual cycle, the differences observed here should not be interpreted as evidence of recurrent seasonal patterns.

The progressive increase in *intI1* towards winter is particularly noteworthy. Class 1 integrons are widely recognized as markers of anthropogenic pollution and are closely associated with the acquisition and dissemination of ARGs [26,27]. Interestingly, the increase in *intI1* was not paralleled by similar trends in *blaCTX-M* or *blaKPC*, suggesting that temporal changes in *intI1* do not necessarily mirror the dynamics of individual resistance genes. This divergence may reflect differences in bacterial hosts, mobility, or ecological distribution of the investigated determinants. It further illustrates the additional information that can be obtained by monitoring *intI1* alongside individual ARGs when investigating AMR dynamics in wastewater.

SG metagenomic sequencing complemented the targeted qPCR analysis by providing a broader characterization of resistome composition and diversity. While qPCR allowed sensitive quantification and temporal monitoring of selected clinically relevant ARGs, SG metagenomics provided a broader overview of resistance classes and gene families. The differences observed between the two approaches therefore reflect their distinct analytical scope, and their combined use provides complementary information. This interpretation is supported by direct methodological comparisons in wastewater surveillance. Knight et al. (2024) showed that high-throughput qPCR provided greater sensitivity for clinically relevant ARGs occurring at low abundance, whereas metagenomic sequencing substantially expanded resistome coverage [13]. Elbait et al. (2024) similarly highlighted methodological differences affecting ARG quantification between qPCR and metagenomic sequencing [12]. Complete concordance between targeted and untargeted approaches should therefore not necessarily be expected at the individual-gene level.

At the broader resistome level, the investigated samples showed broadly similar profiles across sites and sampling periods, with MLS, aminoglycoside, β-lactam, and fluoroquinolone resistance genes consistently representing major components of the wastewater resistome. Comparable compositions have been reported in geographically distinct wastewater systems. Metagenomic investigations in Sicily identified macrolide, β-lactam, and aminoglycoside resistance among the predominant ARG categories [28], while studies conducted in South Africa and other geographical settings similarly reported broad representation of these resistance classes [9,11]. However, the relative contribution of other resistance determinants varied among studies, indicating that wastewater resistome composition is not universally uniform. Such differences may reflect local antimicrobial use, population and wastewater characteristics, bacterial community structure, as well as differences in sequencing depth and bioinformatic workflows. Thus, rather than indicating a universally conserved wastewater resistome, our findings suggest that some major resistance classes are recurrently represented in municipal wastewater, while their relative contribution may vary according to local and methodological factors.

Importantly, the broadly similar resistome profiles observed across the investigated WWTPs should be interpreted as reflecting the widespread occurrence of ARGs in untreated municipal wastewater entering the treatment plants, rather than as evidence that the WWTPs themselves act as AMR reservoirs. Since only influent wastewater was analyzed, the present study cannot assess ARG persistence or removal during wastewater treatment. Paired analyses of influent, intermediate treatment stages, and effluent wastewater would be required to assess ARG persistence and removal throughout the treatment process.

At the gene-family level, OXA-like β-lactamases represented the predominant component of the β-lactam resistome, while variant-level analysis revealed extensive diversity within this family. Class D β-lactamases, including OXA-like determinants, have also been reported in metagenomic investigations of municipal wastewater [29]. Although Sekizuka et al. analyzed treated effluents rather than untreated influent wastewater, the frequent detection of *blaOXA* and *blaGES* and the comparatively limited representation of *blaCTX-M* illustrate the heterogeneous distribution of β-lactamase families across wastewater systems. However, detection of OXA-like genes should not be directly interpreted as evidence of clinically relevant carbapenem resistance, as the OXA family encompasses enzymes with heterogeneous substrate profiles and ecological distributions.

The comparison between the two bioinformatic workflows further highlighted the influence of analytical strategy on resistome characterization. AMR++ provided quantitative information on resistance gene families across the major antimicrobial classes, whereas the CZ ID antimicrobial resistance workflow enabled higher-resolution identification of individual ARG variants but detected a smaller subset of resistance determinants. For example, while AMR++ identified CTX-like and KPC-like β-lactamase families, CZ ID did not detect CTX-M-, KPC-, or NDM-type variants. Likewise, *qnrS* was detected in all samples by qPCR, whereas CZ ID identified *qnrS* variants in 34 of 36 samples. These apparent discrepancies should not be interpreted as conflicting results but rather as consequences of differences in analytical objectives, reference databases, and annotation criteria.

Unlike AMR++, which uses read mapping against curated ARG databases for resistome profiling [30], variant-level characterization using the CZ ID antimicrobial resistance workflow and CARD was based on more stringent assignment criteria, particularly for contig-based detections, for which only Perfect matches were retained [31]. Consequently, fragmented genes or ARGs present at low abundance may remain unassigned. This limitation is consistent with wastewater studies showing that selective culture enrichment can improve the detection of low-abundance clinically relevant resistance determinants, including carbapenemase genes [32]. Thus, failure to identify a specific ARG variant by untargeted metagenomics should not necessarily be interpreted as evidence of its absence from the original wastewater sample.

The use of short-read SG sequencing may further contribute to these differences, as fragmented sequences can limit complete gene reconstruction and confident variant assignment [33]. Long-read metagenomic sequencing can provide additional information on the genomic context of ARGs and facilitate their association with bacterial hosts and mobile genetic elements. In wastewater systems, Dai et al. (2022) demonstrated the value of long-read sequencing for resolving associations between ARGs and mobile genetic elements across sewage and activated sludge [34]. Integration of short- and long-read approaches may therefore further improve the characterization of wastewater resistomes.

Some limitations should be acknowledged. The study covered a single annual cycle and included only untreated influent wastewater; therefore, the observed temporal differences cannot establish recurrent seasonal patterns across years, nor can the study assess ARG persistence or removal during wastewater treatment. Longitudinal metagenomic monitoring was restricted to two representative WWTPs, whereas the remaining plants were investigated during two seasonal surveys. Furthermore, qPCR targeted only a limited number of clinically relevant ARGs, and neither antibiotic consumption data nor clinical or phenotypic resistance data were available for correlation with the molecular findings. Consequently, the potential contribution of these factors to the temporal variations observed in wastewater could not be directly assessed. Detailed information on potential livestock and agricultural contributions within the investigated catchment areas was also unavailable. Future studies integrating multi-year monitoring, different wastewater treatment stages, broader molecular characterization, and epidemiological and catchment-level data could provide a more comprehensive understanding of AMR dynamics in wastewater within a One Health framework.

Overall, this study demonstrates that wastewater surveillance benefits from the integration of complementary molecular and bioinformatic approaches. Targeted qPCR provides sensitive quantification of predefined clinically relevant ARGs, whereas SG metagenomics substantially expands resistome characterization by identifying resistance classes, gene families, and sequence diversity. Within the metagenomic analyses, AMR++ provided comprehensive resistome profiling, while the CZ ID antimicrobial resistance workflow enabled higher-resolution characterization of individual ARG variants under more stringent annotation criteria. Together, these complementary approaches provide a useful framework for wastewater-based AMR surveillance and support their integration within One Health monitoring strategies.

## 4. Materials and Methods

### 4.1. Sample Collection

Composite 24-h influent wastewater samples were collected from eight wastewater treatment plants (WWTPs; S1–S8) located in central Italy, before any treatment process. Specifically, WWTPs S1–S5 are located in the Abruzzo region, while WWTPs S6–S8 are located in the Molise region. Geographical, demographic, and technical characteristics of each WWTP are reported in Table 1. A total of 96 samples were collected monthly from April 2025 to March 2026. Samples were transported to the laboratory under refrigerated conditions (4 °C) and processed within 24 h of collection. All samples were analyzed by real-time qPCR for the detection and quantification of selected ARGs. SG metagenomic sequencing was performed on a subset of 36 samples to characterize the overall wastewater resistome and ARG diversity. For the longitudinal component, S5 and S6 were selected for monthly metagenomic monitoring (12 samples per WWTP), including one WWTP from each investigated region and representing contrasting population equivalents. S5 had the highest population equivalent among the investigated WWTPs in Abruzzo (270,000 PE), whereas S6 had the lowest among those investigated in Molise (23,000 PE). To complement the longitudinal analysis with a broader spatial assessment, all eight WWTPs were analyzed during two seasonal surveys (July 2025 and January 2026).

### 4.2. DNA Extraction of Samples

For each sample, 50 mL of influent wastewater was filtered through sterile nitrocellulose membranes with a pore size of 0.45 μm (Sigma-Aldrich, St. Louis, MO, USA). For each sampling campaign, one blank sample was filtered. Total DNA was extracted using the DNeasy PowerWater Kit (QIAGEN, Hilden, Germany) according to the manufacturer’s instructions. Each sample yielded 100 μL of DNA extract, which was aliquoted and stored at −20 °C until further analysis.

### 4.3. Real-Time PCR Analysis

Real-time qPCR analyses were performed following the protocol published by the Italian Ministry of Health for the detection and quantification of antibiotic resistance genes in untreated urban wastewater [35]. The protocol is intended for the detection and quantification of ARGs in untreated influent wastewater and supports environmental AMR surveillance within the Italian National Action Plan on Antimicrobial Resistance (PNCAR 2022–2025) using a wastewater-based epidemiology approach.

Analyses were carried out using a CFX96 Touch™ Real-Time PCR Detection System (Bio-Rad Laboratories, Hercules, CA, USA). SYBR Green chemistry was used for the amplification of the 16S rRNA, *qnrS*, *blaKPC*, *blaCTX-M* and *intI1* genes. Amplification performance was assessed by slope values ranging from −3.1 to −3.6, amplification efficiencies between 90% and 110%, and correlation coefficients (R^2^) ≥ 0.98. Each sample was analyzed in duplicate, and no-template controls (NTCs) were included in every run. Melting curve analysis was performed to confirm amplification specificity. Absolute quantification was achieved using standard curves generated from serial dilutions of synthetic DNA standards included in each run, following MIQE recommendations [36].

Results were expressed as gene copies per milliliter of wastewater (copies/mL). Relative abundance was calculated by normalizing ARG copy numbers to 16S rRNA gene copies (ARG/16S).

### 4.4. SG Metagenomics Sequencing and Bioinformatic Analysis

Metagenomic libraries were prepared using the Illumina DNA Prep protocol. The libraries were subjected to quality control using the Agilent TapeStation 4200 System (Agilent Technologies, Santa Clara, CA, USA) with Agilent D5000 HS ScreenTape and reagent kit and the Qubit 2.0 Fluorometer (Thermo Fisher Scientific, Waltham, MA, USA), to verify the size distribution and concentration, respectively. Sequencing was performed on Illumina NextSeq 2000 platform using a P3 XLEAP-SBS 300-cycle kit (2 × 150 bp paired-end reads) (Illumina, Inc. San Diego, CA, USA).

Approximately 50 million paired-end reads were generated for each sample. The Read 1 and Read 2 FASTQ files of each sample were downloaded from the Genpat platform, the Italian National Reference Centre for WGS of microbial pathogens (https://github.com/genpat-it) (accessed on 6 August 2026).

Sequencing reads were analyzed using two complementary bioinformatic workflows. Overall resistome composition was characterized using the AMR++ pipeline (v3.0), which aligns quality-filtered reads against the MEGARes database for ARG identification and quantification. Gene abundance was expressed as hits per input Gbp, a sequencing-depth-normalized metric accounting for differences in library size [30,37].

To compare resistome composition among samples, ARGs were grouped according to antibiotic resistance class, and relative abundances (%) were calculated by dividing the abundance of each resistance class by the total abundance of all detected ARG classes within the corresponding sample. For graphical visualization, resistance classes representing < 1% of the total resistome were grouped into a single category labelled “Others”. The most clinical relevant resistance classes (MLS, aminoglycosides, β-lactams and fluoroquinolones) were subsequently investigated at the gene-family level.

For each resistance class, ARGs were grouped according to their corresponding resistance gene families and gene abundances were expressed as the relative proportion (%) within the corresponding resistance class.

To investigate ARG diversity at the sequence-variant level, metagenomic data were additionally analyzed using the antimicrobial resistance workflow implemented in the Chan Zuckerberg ID (CZ ID) platform, which performs ARG annotation against the Comprehensive Antibiotic Resistance Database (CARD) (https://czid.org) [31,38]. For downstream analyses, ARG variants were considered detected when supported by a numerical value in the Reads column. If no read count was reported, only variants identified in the Contigs column and classified as “Perfect” according to the CARD annotation criteria implemented in CZ ID were retained. Variants classified as “Strict” or “Nudged” were excluded from further analyses. For each ARG family, the number of detected variants, their prevalence and the number of positive samples were subsequently determined.

### 4.5. Statistical Analysis

Statistical analyses were performed using GraphPad Prism version 11.0.2 (GraphPad Software, Boston, MA, USA). Relative abundances obtained by qPCR (ARG/16S) were log 10 transformed prior to statistical analysis and median values were calculated for each sampling month and used for temporal comparisons. Temporal differences among monthly sampling periods were assessed using the Kruskal–Wallis test, followed by pairwise comparisons using the Mann–Whitney U test when appropriate. For seasonal analyses, sampling months were grouped as follows: spring (April–June 2025), summer (July–September 2025), fall (October–December 2025), and winter (January–March 2026). Seasonal differences in relative ARG abundance were assessed using the Friedman test to account for repeated sampling of the same WWTPs over time, with WWTP considered as the repeated-measures unit. For each WWTP, the median relative abundance of each ARG was calculated for each season from the corresponding monthly measurements. Statistical significance was set at *p* < 0.05.

## 5. Conclusions

This study demonstrates the value of municipal wastewater as a population-level matrix for monitoring antimicrobial resistance circulating within the community. The integration of targeted qPCR with SG metagenomic sequencing provided complementary information for wastewater-based AMR surveillance, combining the high sensitivity of qPCR for the detection and quantification of selected clinically relevant ARGs with the broader resistome characterization provided by metagenomics. While qPCR enabled quantitative monitoring of predefined resistance markers, SG metagenomics expanded the analysis to resistance classes, gene families, and sequence variants that cannot be captured using targeted assays alone. Together, these complementary approaches provide a useful framework for environmental AMR surveillance and support their integration within One Health monitoring strategies.

## Figures and Tables

**Table 1:** Characteristics of the eight municipal WWTPs included in this study, including site code, wastewater treatment plant, municipality, region, population equivalent (PE) and geographic setting.

Site Code	Wastewater Treatment Plant	Municipality	Region	Population Equivalent (PE)	Geographic Setting
S1	Pile	L’Aquila	Abruzzo	28,000	Inland
S2	Villa Pavone	Teramo	Abruzzo	40,000	Inland
S3	Montesilvano	Montesilvano	Abruzzo	105,000	Coastal
S4	San Martino	Chieti	Abruzzo	18,000	Inland
S5	Pescara	Pescara	Abruzzo	270,000	Coastal
S6	Pantano Basso	Termoli	Molise	23,000	Coastal
S7	Zona Porto	Termoli	Molise	30,000	Coastal
S8	San Pietro	Campobasso	Molise	50,000	Inland

## Data Availability

The original contributions presented in this study are included in the article; further inquiries can be directed to the corresponding author.

## Supplementary Materials

The following supporting information can be downloaded at: https://www.mdpi.com/article/10.3390/antibiotics15090850/s1.

## References

[1] World Health Organization (2022). WHO Strategic Priorities on Antimicrobial Resistance: Preserving Antimicrobials for Today and Tomorrow.

[2] Berendonk T.U., Manaia C.M., Merlin C., Fatta-Kassinos D., Cytryn E., Walsh F., Bürgmann H., Sørum H., Norström M., Pons M.N. (2015). Tackling antibiotic resistance: The environmental framework. Nat. Rev. Microbiol..

[3] European Parliamentary Research Service (2023). Stepping Up EU Action to Combat Antimicrobial Resistance: The “One Health” Approach.

[4] Nguyen A.Q., Vu H.P., Nguyen L.N., Wang Q., Djordjevic S.P., Donner E., Yin H., Nghiem L.D. (2021). Monitoring antibiotic resistance genes in wastewater treatment: Current strategies and future challenges. Sci. Total Environ..

[5] Manaia C.M., Rocha J., Scaccia N., Marano R., Radu E., Biancullo F., Cerqueira F., Fortunato G., Iakovides I.C., Zammit I. (2018). Antibiotic resistance in wastewater treatment plants: Tackling the black box. Environ. Int..

[6] Kalli M., Noutsopoulos C., Mamais D. (2023). The fate and occurrence of antibiotic-resistant bacteria and antibiotic resistance genes during advanced wastewater treatment and disinfection: A review. Water.

[7] Rizzo L., Manaia C., Merlin C., Schwartz T., Dagot C., Ploy M.C., Michael I., Fatta-Kassinos D. (2013). Urban wastewater treatment plants as hotspots for antibiotic resistant bacteria and genes spread into the environment: A review. Sci. Total Environ..

[8] Krzeminski P., Tomei M.C., Karaolia P., Langenhoff A., Almeida C.M.R., Felis E., Gritten F., Andersen H.R., Fernandes T., Manaia C.M. (2019). Performance of secondary wastewater treatment methods for the removal of contaminants of emerging concern implicated in crop uptake and antibiotic resistance spread: A review. Sci. Total Environ..

[9] Hendriksen R.S., Munk P., Njage P., Van Bunnik B., McNally L., Lukjancenko O., Röder T., Nieuwenhuijse D., Pedersen S.K., Kjeldgaard J. (2019). Global monitoring of antimicrobial resistance based on metagenomics analyses of urban sewage. Nat. Commun..

[10] Javvadi Y., Mohan S.V. (2023). Understanding the distribution of antibiotic resistance genes in an urban community using a wastewater-based epidemiological approach. Sci. Total Environ..

[11] Smith A.M., Ramudzulu M., Munk P., Avot B.J., Esterhuyse K.C., Van Blerk N., Kwenda S., Sekwadi P. (2024). Metagenomics analysis of sewage for surveillance of antimicrobial resistance in South Africa. PLoS ONE.

[12] Elbait G.D., Daou M., Abuoudah M., Elmekawy A., Hasan S.W., Everett D.B., Alsafar H., Henschel A., Yousef A.F. (2024). Comparison of qPCR and metagenomic sequencing methods for quantifying antibiotic resistance genes in wastewater. PLoS ONE.

[13] Knight M.E., Webster G., Perry W.B., Baldwin A., Rushton L., Pass D.A., Cross G., Durance I., Muziasari W., Kille P. (2024). National-scale antimicrobial resistance surveillance in wastewater: A comparative analysis of HT qPCR and metagenomic approaches. Water Res..

[14] Caucci S., Karkman A., Cacace D., Rybicki M., Timpel P., Voolaid V., Gurke R., Virta M., Berendonk T.U. (2016). Seasonality of antibiotic prescriptions for outpatients and resistance genes in sewers and wastewater treatment plant outflow. FEMS Microbiol. Ecol..

[15] Guruge S.K., Han Z., Dai S., Islam A., Ben W., Tian Z., Zhang Y., Yang M. (2025). Seasonal variation of antibiotic resistance genes in activated sludge of a full-scale municipal wastewater treatment plant: Contribution of activated sludge functional taxa and clinically relevant taxa. Water Res..

[16] Malcom H.B., Bowes D.A. (2025). Use of wastewater to monitor antimicrobial resistance trends in communities and implications for wastewater-based epidemiology: A review of the recent literature. Microorganisms.

[17] Bonanno Ferraro G., Bonomo C., Brandtner D., Mancini P., Veneri C., Briancesco R., Coccia A.M., Lucentini L., Suffredini E., Bongiorno D. (2024). Characterisation of microbial communities and quantification of antibiotic resistance genes in Italian wastewater treatment plants using 16S rRNA sequencing and digital PCR. Sci. Total Environ..

[18] Corno G., Yang Y., Eckert E.M., Fontaneto D., Fiorentino A., Galafassi S., Zhang T., Di Cesare A. (2019). Effluents of wastewater treatment plants promote the rapid stabilization of the antibiotic resistome in receiving freshwater bodies. Water Res..

[19] Bonetta S., Di Cesare A., Pignata C., Sabatino R., Macrì M., Corno G., Panizzolo M., Bonetta S., Carraro E. (2023). Occurrence of antibiotic-resistant bacteria and resistance genes in the urban water cycle. Environ. Sci. Pollut. Res..

[20] Di Cesare A., Eckert E.M., D’Urso S., Bertoni R., Gillan D.C., Wattiez R., Corno G. (2016). Co-occurrence of integrase 1, antibiotic and heavy metal resistance genes in municipal wastewater treatment plants. Water Res..

[21] Magnano San Lio R., Maugeri A., Barchitta M., Favara G., La Rosa M.C., La Mastra C., Agodi A. (2025). Monitoring antibiotic resistance in wastewater: Findings from three treatment plants in Sicily, Italy. Int. J. Environ. Res. Public Health.

[22] Petricciuolo M., Carnevali A., Torboli A., Postinghel M., Guasticchi A., Foladori P., Cadonna M., Federici E. (2026). Wastewater-based assessment of antimicrobial resistance and bacterial communities in urban and rural areas in the Province of Trento (Italy). MicrobiologyOpen.

[23] Macrì M., Bonetta S., Di Cesare A., Sabatino R., Corno G., Catozzo M., Pignata C., Mecarelli E., Medana C., Carraro E. (2024). Antibiotic resistance and pathogen spreading in a wastewater treatment plant designed for wastewater reuse. Environ. Pollut..

[24] Silvester R., Woodhall N., Nurmi W., Muziasari W., Farkas K., Cross G., Malham S.K., Jones D.L. (2025). High-throughput qPCR profiling of antimicrobial resistance genes and bacterial loads in wastewater and receiving environments. Environ. Pollut..

[25] Freitas J.F., Oliveira T.T., Agnez-Lima L.F. (2026). Longitudinal wastewater metagenomics reveals distinct environmental and anthropogenic associations with resistance, virulence, and viral communities. Environ. Pollut..

[26] Gillings M.R., Gaze W.H., Pruden A., Smalla K., Tiedje J.M., Zhu Y.G. (2015). Using the class 1 integron-integrase gene as a proxy for anthropogenic pollution. ISME J..

[27] Guo J., Li J., Chen H., Bond P.L., Yuan Z. (2017). Metagenomic analysis reveals wastewater treatment plants as hotspots of antibiotic resistance genes and mobile genetic elements. Water Res..

[28] Magnano San Lio R., Maugeri A., Barchitta M., Favara G., La Rosa M.C., La Mastra C., Ferrante M., Agodi A. (2026). The wastewater resistome: A shotgun metagenomics analysis of urban treatment plants in Sicily. Antibiotics.

[29] Sekizuka T., Itokawa K., Tanaka R., Hashino M., Yatsu K., Kuroda M. (2022). Metagenomic analysis of urban wastewater treatment plant effluents in Tokyo. Infect. Drug Resist..

[30] Bonin N., Doster E., Worley H., Pinnell L.J., Bravo J.E., Ferm P., Marini S., Prosperi M., Noyes N., Morley P.S. (2023). MEGARes and AMR++, v3.0: An updated comprehensive database of antimicrobial resistance determinants and an improved software pipeline for classification using high-throughput sequencing. Nucleic Acids Res..

[31] Lu D., Kalantar K.L., Glascock A.L., Chu V.T., Guerrero E.S., Bernick N., Butcher X., Ewing K., Fahsbender E., Holmes O. (2025). Simultaneous detection of pathogens and antimicrobial resistance genes with the open-source, cloud-based CZ ID platform. Genome Med..

[32] Acosta N., Lee J., Bautista M.A., Bhatnagar S., Li C., Waddell B.J., Au E., Pradhan P., Clark R.G., Meddings J. (2025). Metagenomic analysis after selective culture enrichment of hospital and community wastewater enhances antimicrobial resistance gene detection. mBio.

[33] Boolchandani M., D’Souza A.W., Dantas G. (2019). Sequencing-based methods and resources to study antimicrobial resistance. Nat. Rev. Genet..

[34] Dai D., Brown C., Bürgmann H., Larsson D.G.J., Nambi I., Zhang T., Flach C.F., Pruden A., Vikesland P.J. (2022). Long-read metagenomic sequencing reveals shifts in associations of antibiotic resistance genes with mobile genetic elements from sewage to activated sludge. Microbiome.

[35] Federici E., Petricciuolo M., La Rosa G., Bonanno Ferraro G., GdL Geni di Antibiotico Resistenza (2025). Ricerca e Quantificazione di Geni di Antibiotico Resistenza nei Reflui Urbani non Trattati.

[36] Bustin S.A., Benes V., Garson J.A., Hellemans J., Huggett J., Kubista M., Mueller R., Nolan T., Pfaffl M.W., Shipley G.L. (2009). The MIQE guidelines: Minimum information for publication of quantitative real-time PCR experiments. Clin. Chem..

[37] Lakin S.M., Dean C., Noyes N.R., Dettenwanger A., Ross A.S., Doster E., Rovira P., Abdo Z., Jones K.L., Ruiz J. (2017). MEGARes: An antimicrobial resistance database for high-throughput sequencing. Nucleic Acids Res..

[38] Kalantar K.L., Carvalho T., de Bourcy C.F.A., Dimitrov B., Dingle G., Egger R., Han J., Holmes O.B., Juan Y.F., King R. (2020). IDseq—An open-source, cloud-based pipeline and analysis service for metagenomic pathogen detection and monitoring. GigaScience.

