# Supplementary material for: Integrated qPCR and Shotgun Metagenomics for Surveillance of Antimicrobial Resistance in Municipal Wastewater from Central Italy
Source: Antibiotics (Basel). 2026 Aug 31;15(9):850. doi: 10.3390/antibiotics15090850 (PMC13603271; doi:10.3390/antibiotics15090850)

Supplementary Figure S1. Temporal and spatial profiles of antimicrobial resistance gene (ARG) classes in municipal wastewater based on shotgun metagenomic sequencing (AMR++ pipeline). (A–B) Temporal distribution of ARG classes at WWTPs S5 and S6 from April 2025 to March 2026. (C) Spatial comparison of ARG class composition among the eight WWTPs sampled in July 2025 and January 2026. Relative abundances are expressed as the percentage of total ARG abundance (hits per input Gbp). ARG classes representing <1% of the total resistome were grouped as "Others".

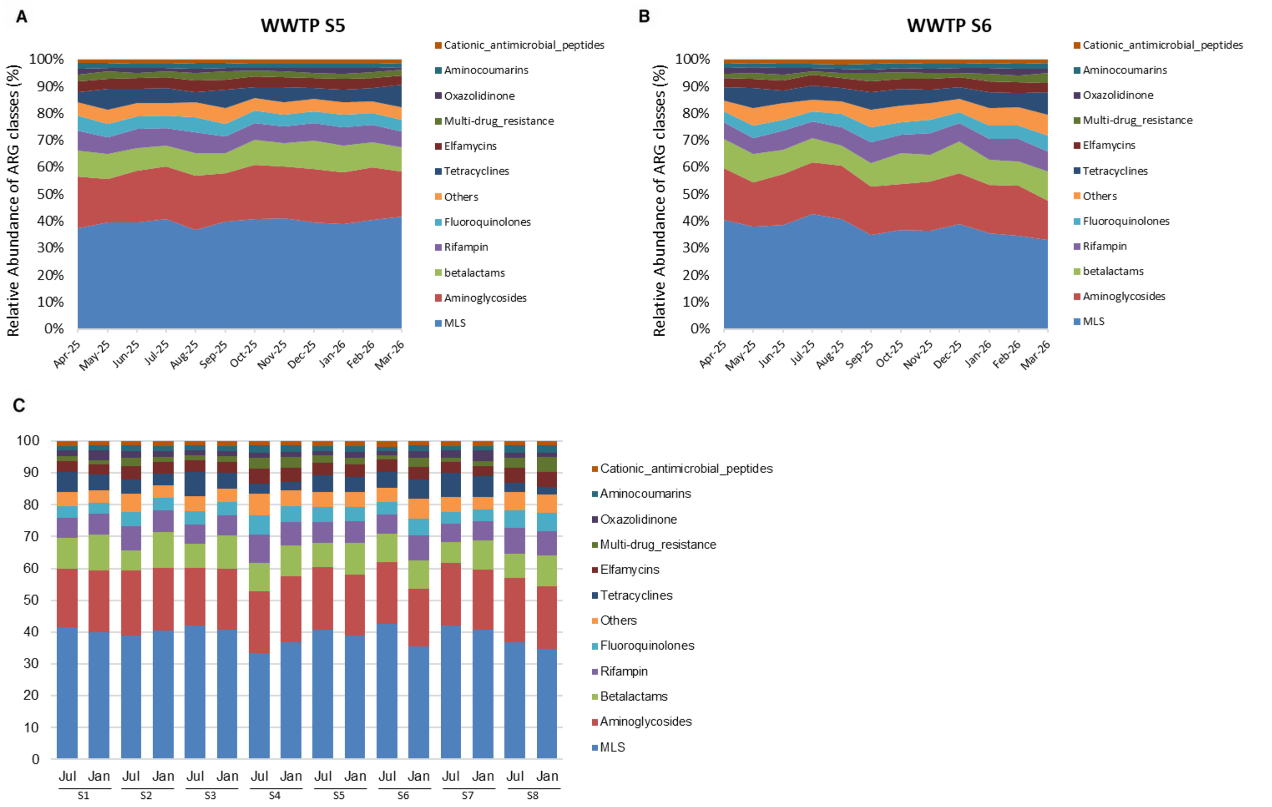

Supplement: Supplementary file 1 [file antibiotics-15-00850-s001.zip › Supplementary_figure.pdf]
