# Supplementary material for: Integrated qPCR and Shotgun Metagenomics for Surveillance of Antimicrobial Resistance in Municipal Wastewater from Central Italy
Source: Antibiotics (Basel). 2026 Aug 31;15(9):850. doi: 10.3390/antibiotics15090850 (PMC13603271; doi:10.3390/antibiotics15090850)
Supplement: Supplementary file 1 [file antibiotics-15-00850-s001.zip › Supplementary_tables.pdf]

Supplementary Table S1. Absolute abundance (gene copies/mL) and relative abundance (gene copies normalized to the 16S rRNA gene) of *qnrS*, *blaKPC*, *blaCTX-M*, and *intI1* quantified by qPCR in wastewater samples collected from the eight wastewater treatment plants during the study period.

		**Absolute abundance (gc/ml)**					**Relative abundance (gc ARG/ gc 16S rRNA)**			
**Sampling time**	**WWTP**	**16S rRNA**	***qnrS***	***blaKPC***	***blaCTX-M***	***intI1***	***qnrS***	***blaKPC***	***blaCTX-M***	***intI1***
April 2025	S1	3,11E+08	7,17E+06	2,84E+03	3,34E+04	1,20E+06	2,31E-02	9,13E-06	1,08E-04	3,87E-03
April 2025	S2	2,85E+08	5,05E+05	2,32E+03	5,05E+03	3,40E+06	1,77E-03	8,15E-06	1,77E-05	1,19E-02
April 2025	S3	5,17E+07	1,72E+04	4,03E+02	6,59E+02	7,45E+04	3,32E-04	7,78E-06	1,27E-05	1,44E-03
April 2025	S4	2,31E+08	1,70E+06	1,96E+03	5,59E+03	6,44E+05	7,38E-03	8,46E-06	2,42E-05	2,79E-03
April 2025	S5	1,67E+08	3,13E+05	3,80E+03	3,37E+03	4,57E+05	1,87E-03	2,27E-05	2,02E-05	2,73E-03
April 2025	S6	1,81E+08	6,46E+05	2,31E+04	1,10E+04	5,19E+05	3,56E-03	1,27E-04	6,07E-05	2,86E-03
April 2025	S7	1,81E+08	1,38E+06	1,13E+04	9,88E+03	5,50E+05	7,61E-03	6,27E-05	5,46E-05	3,04E-03
April 2025	S8	5,30E+07	7,98E+03	1,63E+02	7,34E+01	7,71E+04	1,51E-04	3,07E-06	1,39E-06	1,46E-03
May 2025	S1	2,30E+08	3,64E+05	1,25E+03	3,02E+03	2,71E+05	1,58E-03	5,45E-06	1,31E-05	1,18E-03
May 2025	S2	1,40E+08	1,31E+06	1,73E+03	7,60E+03	3,59E+05	9,36E-03	1,24E-05	5,44E-05	2,57E-03
May 2025	S3	4,31E+07	2,63E+05	2,90E+03	1,75E+03	1,69E+05	6,10E-03	6,74E-05	4,06E-05	3,93E-03
May 2025	S4	2,10E+08	2,45E+06	5,53E+04	3,69E+04	4,57E+05	1,17E-02	2,64E-04	1,76E-04	2,18E-03
May 2025	S5	1,62E+08	4,14E+05	4,65E+03	3,07E+03	3,96E+05	2,56E-03	2,88E-05	1,90E-05	2,45E-03
May 2025	S6	3,28E+08	1,26E+06	1,54E+04	1,65E+04	3,57E+05	3,85E-03	4,71E-05	5,02E-05	1,09E-03
May 2025	S7	1,77E+08	4,40E+05	4,22E+03	1,40E+04	2,69E+05	2,48E-03	2,38E-05	7,87E-05	1,52E-03
May 2025	S8	7,99E+07	3,36E+05	2,40E+03	4,90E+03	1,05E+05	4,20E-03	3,00E-05	6,13E-05	1,32E-03
June 2025	S1	2,57E+08	5,10E+05	1,72E+05	7,32E+03	1,75E+05	1,98E-03	6,68E-04	2,85E-05	6,80E-04
June 2025	S2	1,76E+08	8,74E+05	3,06E+04	1,58E+04	3,26E+05	4,95E-03	1,74E-04	8,97E-05	1,85E-03
June 2025	S3	3,24E+08	2,27E+06	4,79E+03	1,96E+04	2,59E+05	7,01E-03	1,48E-05	6,04E-05	7,98E-04
June 2025	S4	1,62E+08	2,09E+05	6,08E+03	7,59E+02	2,31E+05	1,29E-03	3,76E-05	4,69E-06	1,43E-03
June 2025	S5	2,10E+08	5,29E+05	1,41E+04	1,68E+04	8,15E+04	2,52E-03	6,72E-05	8,00E-05	3,89E-04
June 2025	S6	3,68E+08	1,65E+06	3,71E+04	3,93E+04	4,40E+05	4,47E-03	1,01E-04	1,07E-04	1,20E-03
June 2025	S7	3,21E+08	3,96E+05	2,43E+03	9,82E+03	3,14E+05	1,23E-03	7,58E-06	3,06E-05	9,77E-04
June 2025	S8	7,16E+07	1,39E+05	2,86E+03	1,30E+03	1,34E+05	1,94E-03	4,00E-05	1,81E-05	1,87E-03
July 2025	S1	1,04E+08	8,19E+04	2,99E+03	4,10E+02	1,54E+05	7,85E-04	2,86E-05	3,93E-06	1,47E-03
July 2025	S2	2,99E+08	1,17E+06	2,25E+04	1,04E+04	1,09E+06	3,89E-03	7,50E-05	3,48E-05	3,64E-03
July 2025	S3	2,18E+08	1,77E+05	9,66E+02	1,04E+03	3,64E+05	8,14E-04	4,44E-06	4,77E-06	1,67E-03
July 2025	S4	2,97E+08	5,03E+05	1,64E+04	7,47E+03	5,91E+05	1,70E-03	5,54E-05	2,52E-05	1,99E-03
July 2025	S5	3,10E+08	1,55E+06	3,26E+03	1,01E+04	7,32E+05	5,00E-03	1,05E-05	3,27E-05	2,36E-03
July 2025	S6	3,08E+08	9,93E+05	9,32E+04	2,24E+04	4,14E+05	3,22E-03	3,02E-04	7,28E-05	1,34E-03
July 2025	S7	2,17E+08	7,40E+05	7,51E+04	1,69E+04	5,35E+05	3,41E-03	3,45E-04	7,79E-05	2,46E-03
July 2025	S8	5,80E+08	2,25E+06	1,10E+06	1,88E+04	7,62E+05	3,88E-03	1,89E-03	3,25E-05	1,32E-03
August 2025	S1	1,54E+07	6,19E+04	9,68E+01	6,38E+02	4,20E+05	4,02E-03	6,29E-06	4,14E-05	2,73E-02
August 2025	S2	7,78E+06	1,04E+04	6,15E+01	4,97E+02	3,53E+05	1,34E-03	7,90E-06	6,39E-05	4,53E-02
August 2025	S3	6,88E+06	1,10E+04	1,85E+02	5,64E+02	3,20E+05	1,60E-03	2,68E-05	8,20E-05	4,65E-02
August 2025	S4	3,87E+07	1,56E+04	3,73E+02	8,54E+02	3,81E+05	4,03E-04	9,64E-06	2,21E-05	9,84E-03
August 2025	S5	1,68E+07	2,72E+04	7,57E+02	8,73E+02	5,64E+05	1,62E-03	4,51E-05	5,20E-05	3,36E-02
August 2025	S6	2,11E+07	6,04E+04	4,90E+01	1,07E+03	4,24E+05	2,87E-03	2,33E-06	5,10E-05	2,01E-02
August 2025	S7	2,63E+07	1,91E+05	5,70E+02	1,82E+03	7,83E+05	7,27E-03	2,17E-05	6,93E-05	2,98E-02
August 2025	S8	1,06E+07	1,21E+04	5,09E+01	4,56E+02	1,92E+05	1,13E-03	4,78E-06	4,29E-05	1,80E-02
September 2025	S1	2,70E+07	1,19E+05	1,13E+02	3,59E+02	9,01E+05	4,42E-03	4,17E-06	1,33E-05	3,34E-02
September 2025	S2	3,59E+07	2,09E+04	1,58E+02	5,59E+01	1,12E+06	5,82E-04	4,41E-06	1,56E-06	3,13E-02
September 2025	S3	3,22E+07	6,30E+04	1,70E+03	9,80E+02	1,02E+06	1,96E-03	5,29E-05	3,05E-05	3,18E-02
September 2025	S4	3,44E+07	3,68E+03	1,20E+02	1,39E+01	1,93E+05	1,07E-04	3,49E-06	4,05E-07	5,62E-03
September 2025	S5	3,71E+07	8,77E+04	7,76E+02	2,16E+02	1,26E+06	2,36E-03	2,09E-05	5,83E-06	3,40E-02
September 2025	S6	2,23E+07	4,33E+04	7,04E+01	7,93E+02	7,67E+05	1,94E-03	3,16E-06	3,56E-05	3,44E-02
September 2025	S7	6,45E+06	1,97E+05	1,75E+02	3,63E+02	1,48E+06	3,05E-02	2,71E-05	5,63E-05	2,29E-01
September 2025	S8	1,09E+07	1,29E+04	5,05E+01	8,75E+01	3,09E+05	1,18E-03	4,62E-06	8,02E-06	2,83E-02
October 2025	S1	4,12E+07	1,59E+05	1,39E+02	2,56E+02	8,73E+05	3,85E-03	3,36E-06	6,21E-06	2,12E-02
October 2025	S2	2,83E+07	5,91E+04	1,34E+02	1,38E+02	6,76E+05	2,09E-03	4,73E-06	4,87E-06	2,39E-02
October 2025	S3	1,50E+07	5,25E+04	1,82E+02	8,04E+02	6,51E+05	3,50E-03	1,21E-05	5,36E-05	4,34E-02
October 2025	S4	7,67E+06	2,48E+03	1,54E+01	9,13E+00	1,66E+05	3,24E-04	2,01E-06	1,19E-06	2,16E-02
October 2025	S5	3,20E+07	9,29E+04	4,86E+02	1,03E+03	1,03E+06	2,90E-03	1,52E-05	3,22E-05	3,20E-02
October 2025	S6	2,36E+07	8,54E+04	6,22E+01	1,03E+03	8,01E+05	3,62E-03	2,64E-06	4,37E-05	3,40E-02

October 2025	S7	7,65E+06	5,37E+04	2,37E+01	9,58E+01	5,88E+05	7,02E-03	3,10E-06	1,25E-05	7,68E-02
October 2025	S8	2,18E+07	1,93E+04	3,14E+01	5,01E+01	5,90E+05	8,87E-04	1,44E-06	2,31E-06	2,71E-02
November 2025	S1	5,93E+07	1,26E+05	1,71E+02	2,00E+03	1,27E+06	2,13E-03	2,88E-06	3,37E-05	2,14E-02
November 2025	S2	1,11E+08	1,52E+05	1,37E+03	2,05E+03	2,25E+06	1,37E-03	1,24E-05	1,85E-05	2,04E-02
November 2025	S3	7,02E+07	1,91E+05	2,11E+03	2,19E+03	1,88E+06	2,73E-03	3,01E-05	3,12E-05	2,68E-02
November 2025	S4	5,88E+07	3,29E+04	5,49E+02	1,63E+02	1,12E+06	5,60E-04	9,34E-06	2,77E-06	1,90E-02
November 2025	S5	4,55E+07	7,59E+04	4,29E+02	4,95E+02	1,16E+06	1,67E-03	9,43E-06	1,09E-05	2,54E-02
November 2025	S6	3,07E+09	1,88E+05	3,35E+02	1,45E+03	2,30E+06	6,12E-05	1,09E-07	4,73E-07	7,49E-04
November 2025	S7	3,99E+07	7,78E+04	1,43E+02	4,75E+02	1,21E+06	1,95E-03	3,60E-06	1,19E-05	3,04E-02
November 2025	S8	6,05E+07	3,16E+04	7,89E+01	1,91E+02	1,30E+06	5,23E-04	1,30E-06	3,16E-06	2,14E-02
December 2025	S1	4,77E+08	1,08E+05	1,65E+02	9,61E+02	1,30E+06	2,25E-04	3,45E-07	2,01E-06	2,72E-03
December 2025	S2	2,50E+08	4,33E+05	5,33E+02	2,04E+03	3,79E+06	1,73E-03	2,13E-06	8,16E-06	1,51E-02
December 2025	S3	2,01E+08	1,43E+05	4,00E+03	1,13E+03	2,10E+06	7,10E-04	1,99E-05	5,63E-06	1,04E-02
December 2025	S4	1,54E+07	3,92E+03	4,06E+01	1,09E+01	3,04E+05	2,54E-04	2,63E-06	7,09E-07	1,98E-02
December 2025	S5	4,14E+07	1,03E+05	1,09E+03	5,96E+02	1,35E+06	2,49E-03	2,62E-05	1,44E-05	3,26E-02
December 2025	S6	3,26E+07	1,11E+05	8,34E+01	6,02E+02	7,35E+05	3,40E-03	2,56E-06	1,84E-05	2,25E-02
December 2025	S7	1,12E+08	1,80E+05	1,68E+02	1,25E+03	1,33E+06	1,60E-03	1,50E-06	1,11E-05	1,19E-02
December 2025	S8	5,23E+07	2,23E+04	1,93E+02	2,72E+02	6,70E+05	4,27E-04	3,70E-06	5,19E-06	1,28E-02
January 2026	S1	5,05E+07	3,26E+05	1,26E+02	3,86E+02	5,40E+06	6,46E-03	2,49E-06	7,65E-06	1,07E-01
January 2026	S2	3,78E+07	6,52E+05	7,48E+02	4,75E+02	7,56E+06	1,73E-02	1,98E-05	1,26E-05	2,00E-01
January 2026	S3	3,16E+07	4,43E+05	1,13E+03	7,35E+02	4,15E+06	1,40E-02	3,59E-05	2,33E-05	1,31E-01
January 2026	S4	2,09E+07	7,65E+04	4,14E+02	3,46E+01	3,15E+06	3,66E-03	1,98E-05	1,65E-06	1,50E-01
January 2026	S5	2,83E+07	4,64E+05	6,46E+02	2,72E+02	6,88E+06	1,64E-02	2,29E-05	9,62E-06	2,44E-01
January 2026	S6	1,05E+07	2,55E+05	2,06E+01	1,07E+02	2,21E+06	2,42E-02	1,96E-06	1,02E-05	2,10E-01
January 2026	S7	2,26E+07	1,93E+05	2,24E+01	1,84E+02	1,70E+06	8,57E-03	9,95E-07	8,17E-06	7,54E-02
January 2026	S8	1,65E+07	6,09E+03	4,09E+00	1,37E+01	1,49E+06	3,69E-04	2,48E-07	8,32E-07	9,06E-02
February 2026	S1	4,24E+07	1,79E+05	3,06E+02	4,00E+02	3,87E+06	4,23E-03	7,21E-06	9,45E-06	9,14E-02
February 2026	S2	6,74E+07	2,76E+05	5,78E+02	3,83E+02	3,95E+06	4,10E-03	8,57E-06	5,68E-06	5,87E-02
February 2026	S3	2,10E+07	1,86E+05	1,37E+02	1,38E+02	3,69E+06	8,87E-03	6,54E-06	6,56E-06	1,75E-01
February 2026	S4	2,16E+06	6,61E+02	1,44E+01	1,00E+00	3,02E+05	3,06E-04	6,65E-06	4,65E-07	1,40E-01
February 2026	S5	2,82E+07	3,33E+05	4,30E+02	1,89E+02	3,69E+06	1,18E-02	1,52E-05	6,71E-06	1,31E-01
February 2026	S6	1,42E+07	2,27E+05	4,17E+01	1,50E+02	3,63E+06	1,61E-02	2,94E-06	1,06E-05	2,56E-01
February 2026	S7	3,20E+07	1,39E+05	5,35E+01	1,73E+02	2,85E+06	4,35E-03	1,67E-06	5,40E-06	8,89E-02
February 2026	S8	2,45E+07	1,24E+04	5,09E+00	3,35E+01	1,75E+06	5,07E-04	2,08E-07	1,37E-06	7,15E-02
March 2026	S1	1,02E+08	1,39E+05	5,20E+02	1,78E+02	1,85E+06	1,72E-04	9,87E-06	1,33E-07	4,48E-03
March 2026	S2	1,23E+08	5,02E+05	3,06E+02	1,35E+04	2,16E+06	2,45E-03	9,24E-06	9,14E-06	1,56E-02
March 2026	S3	7,87E+07	1,75E+05	1,02E+02	2,95E+04	2,19E+06	4,91E-03	1,09E-06	6,42E-06	4,46E-02
March 2026	S4	1,09E+08	4,06E+04	4,58E+02	2,72E+04	3,35E+06	1,36E-03	4,50E-06	1,75E-06	1,82E-02
March 2026	S5	1,08E+08	1,73E+05	3,10E+01	3,93E+05	2,35E+06	3,71E-04	2,83E-07	2,49E-04	3,06E-02
March 2026	S6	9,37E+07	4,60E+05	7,20E+01	6,02E+02	4,18E+06	2,23E-03	9,15E-07	3,74E-04	2,78E-02
March 2026	S7	3,31E+07	8,13E+04	8,00E+00	3,03E+02	5,18E+05	1,60E-03	7,40E-08	3,63E-03	2,18E-02
March 2026	S8	5,27E+07	9,08E+03	2,21E+02	7,03E+00	2,36E+05	4,09E-03	1,80E-06	1,10E-04	1,76E-02

Supplementary Table S2. Metadata and sequencing statistics for the 36 wastewater samples analysed by shotgun metagenomic sequencing, including sample information, sequencing output and the number and percentage of reads assigned to antimicrobial resistance (AMR) genes.

Sample_id	Sample_type	Collection year	Collection month	Total reads	Total gbp	Total reads AMR	% reads AMR
S1	wastewater	2025	7	44.965.596	6,699733282	545957	1,214166048
S1	wastewater	2026	1	45.907.484	6,838137512	572863	1,247864074
S2	wastewater	2025	7	47.315.622	7,053099102	451560	0,954357104
S2	wastewater	2026	1	43.562.166	6,487671724	547838	1,25760046
S3	wastewater	2025	7	46.360.140	6,914763162	474021	1,022475342
S3	wastewater	2026	1	57.941.564	8,641836334	646237	1,115325434
S4	wastewater	2025	7	45.434.420	6,77163538	287055	0,631800736
S4	wastewater	2026	1	49.192.428	7,331329238	373330	0,758917612
S5	wastewater	2025	4	46.092.654	6,873801872	407668	0,884453301
S5	wastewater	2025	5	20.501.844	3,04451294	180176	0,878828265
S5	wastewater	2025	6	62.974.528	9,402503534	538394	0,854939318
S5	wastewater	2025	7	71.262.550	10,63749084	745310	1,045864904
S5	wastewater	2025	8	30.361.888	4,521043126	292662	0,963912389
S5	wastewater	2025	9	26.288.496	3,905708368	223184	0,848979721
S5	wastewater	2025	10	21.031.236	3,127708564	222752	1,059148402
S5	wastewater	2025	11	34.433.718	5,132776092	340004	0,987415881
S5	wastewater	2025	12	58.607.306	8,749343248	646469	1,103051896
S5	wastewater	2026	1	51.918.634	7,749202592	483571	0,931401624
S5	wastewater	2026	2	39.844.612	5,939788994	415939	1,043902749
S5	wastewater	2026	3	32.864.744	4,89557835	286019	0,870291276
s6	wastewater	2025	4	81.978.616	12,2492081	959950	1,170976099
s6	wastewater	2025	5	16.811.404	2,486664688	164853	0,980602215
s6	wastewater	2025	6	12.896.228	1,904675786	137418	1,065567389
s6	wastewater	2025	7	49.780.244	7,43043939	598114	1,201508775
S6	wastewater	2025	8	56.407.716	8,427665838	532896	0,944721818
S6	wastewater	2025	9	45.850.396	6,833651598	320273	0,698517413
S6	wastewater	2025	10	26.729.914	3,960827314	225403	0,843261224
S6	wastewater	2025	11	50.611.010	7,54773393	414789	0,819562779
S6	wastewater	2025	12	39.623.830	5,90149247	425342	1,073449992
S6	wastewater	2026	1	45.366.302	6,764203178	307964	0,678838668
S6	wastewater	2026	2	45.934.306	6,850785762	288359	0,62776392
S6	wastewater	2026	3	30.822.142	4,588047018	153848	0,499147658
S7	wastewater	2025	7	46.362.598	6,90793131	544609	1,174673171
S7	wastewater	2026	1	23.674.062	3,51833554	296883	1,254043349
S8	wastewater	2025	7	35.671.146	5,314720266	280643	0,786750726
S8	wastewater	2026	1	47.073.542	7,001210914	343896	0,730550508

Supplementary Table S3. Relative abundance of aminoglycoside resistance gene families detected in municipal wastewater samples. Values are reported as the median relative abundance and observed range (minimum–maximum) across all samples included in the temporal monitoring of sites S5 and S6 and in the spatial surveys conducted in July 2025 and January 2026. Gene families with low individual abundance were grouped into the category "Others".

**Gene family**	**Median (%)**	**Range**
ANT(3″)	28,8	11.9–34.7
AAC(6′)	21,0	17.4–27.7
APH(3′)	10,9	8.8–13.4
AAC(3)	7,6	4.5–12.1
APH(6)	7,7	4.5–10.2
ANT(6)	6,0	3.2–11.4
APH(3″)	3,6	1.9–5.8
APH(2″)	2,4	1.5–4.4
GRM	1,1	0.6–2.7
APH(9)	1,0	0.6–1.6
ANT(9)	0,9	0.6–2.0
AAC(2′)	0,9	0.3–1.9
RanA	0,8	0.2–1.9
KamB	0,6	0.3–1.7
Others	6,9	5.0–11.8

Supplementary Table S4. Relative abundance of β-lactam resistance gene families detected in municipal wastewater samples. Values are reported as the observed range (minimum–maximum) across all samples included in the temporal monitoring of sites S5 and S6 and in the spatial surveys conducted in July 2025 and January 2026. Relative abundances were calculated as the proportion of each β-lactamase family within the total β-lactam resistance class. Gene families with low individual abundance were grouped into the category "Others".

**Gene family**	**Median (%)**	**Range**
OXA	50,5	27.02–68.68
GES	2,8	1.07–6.64
FOX	2,6	0.61–4.90
CTX	1,8	0.90–4.43
IMP	0,8	0.37–2.04
SHV	0,5	0.23–1.13
TEM	0,3	0.17–0.48
KPC	0,2	0.11–0.49
VIM	0,1	0.07–0.40
NDM	0,1	0.02–0.20
Others	30,9	14.21–53.35

Supplementary Table S5. Relative abundance of fluoroquinolone resistance gene families detected in municipal wastewater samples. Values are reported as the observed range (minimum–maximum) across all samples included in the temporal monitoring of sites S5 and S6 and in the spatial surveys conducted in July 2025 and January 2026. Relative abundances were calculated as the proportion of each fluoroquinolone resistance gene family within the total fluoroquinolone resistance class. Gene families with low individual abundance were grouped into the category "Others".

**Gene family**	**Median (%)**	**Range**
QEPA	37,60	16.09–63.37
QNRS	21,62	3.17–58.97
QNRB	12,83	5.99–38.47
QNRVC	11,87	3.95–34.38
PATA	2,04	0.50–6.55
QNRA	2,07	0.49–6.80
QNRC	1,82	0.29–11.29
MFPA	1,74	0.00–7.98
QNRD	1,08	0.00–4.84
Others	2,39	0.93–8.38

Supplementary Table S6. Relative abundance of macrolide–lincosamide–streptogramin (MLS) resistance gene families detected in municipal wastewater samples. Values are reported as the observed range (minimum–maximum) across all samples included in the temporal monitoring of sites S5 and S6 and in the spatial surveys conducted in July 2025 and January 2026. Relative abundances were calculated as the proportion of each MLS resistance gene family within the total MLS resistance class. Gene families with low individual abundance were grouped into the category "Others".

**Gene family**	**Median (%)**	**Range**
mph	60,5	14.0–44.7
erm	25,8	28.6–79.4
ere	3,6	1.6–10.5
lnu/lin	4,4	2.5–7.9
vat	1,2	0.5–2.7
vgb	0,2	0.0–0.7
Others	3,1	0.8–10.0

Supplementary Table S7. Diversity and prevalence of clinically relevant β-lactamase families identified by CZ ID workflow in 36 wastewater metagenomes.

**Gene family**	**No. of variants**	**Variants detected**	**Positive samples (n = 36)**
OXA beta-lactamase	109	OXA-1, OXA-2, OXA-3, OXA-4, OXA-5, OXA-9, OXA-10, OXA-12, OXA-17, OXA-20, OXA-21, OXA-31, OXA-37, OXA-45, OXA-46, OXA-47, OXA-56, OXA-58, OXA-96, OXA-97, OXA-101, OXA-118, OXA-119, OXA-129, OXA-155, OXA-161, OXA-164, OXA-198, OXA-204, OXA-205, OXA-209, OXA-210, OXA-211, OXA-212, OXA-276, OXA-280, OXA-281, OXA-296, OXA-299, OXA-309, OXA-333, OXA-334, OXA-347, OXA-372, OXA-373, OXA-392, OXA-415, OXA-420, OXA-427, OXA-464, OXA-491, OXA-498, OXA-504, OXA-512, OXA-514, OXA-520, OXA-534, OXA-539, OXA-541, OXA-543, OXA-544, OXA-565, OXA-641, OXA-643, OXA-644, OXA-645, OXA-650, OXA-651, OXA-652, OXA-656, OXA-662, OXA-667, OXA-676, OXA-681, OXA-724, OXA-726, OXA-728, OXA-732, OXA-737, OXA-779, OXA-780, OXA-795, OXA-796, OXA-824, OXA-827, OXA-835, OXA-896, OXA-912, OXA-917, OXA-928, OXA-932	**36 (100%)**
GES beta-lactamase	20	GES-2, GES-3, GES-6, GES-7, GES-8, GES-9, GES-13, GES-14, GES-16, GES-18, GES-24, GES-26, GES-27, GES-28, GES-29, GES-35, GES-38, GES-44, GES-45, GES-46	**33 (91.6%)**
FOX beta-lactamase	13	FOX-1, FOX-2, FOX-3, FOX-4, FOX-5, FOX-7, FOX-8, FOX-9, FOX-10, FOX-12, FOX-13, FOX-14, FOX-15	**27 (75.0%)**
TEM beta-lactamase	11	TEM-70, TEM-73, TEM-82, TEM-160, TEM-178, TEM-201, TEM-207, TEM-230, TEM-235, TEM-236, TEM-240	**17 (47.2%)**
IMP beta-lactamase	5	IMP-12, IMP-13, IMP-22, IMP-33, IMP-37	**6 (16.7%)**
SHV beta-lactamase	5	SHV-134, SHV-142, SHV-158, SHV-183, SHV-210	**6 (16.7%)**
VIM beta-lactamase	4	VIM-12, VIM-32, VIM-37, VIM-39	**4 (11.1%)**
KPC beta-lactamase	Not detected	-	**0 (0.0%)**
CTX-M beta-lactamase	Not detected	-	**0 (0.0%)**
NDM beta-lactamase	Not detected	-	**0 (0.0%)**

Supplementary Table S8. Diversity and prevalence of clinically relevant Aminoglycosides families identified by CZ ID workflow in 36 wastewater metagenomes.

**Gene family**	**No. of variants**	**Variants detected**	**Positive samples (n = 36)**
ACC	9	AAC(3)-Ia, AAC(3)-IIe, AAC(3)-IId, AAC(6')-Ib, AAC(6')-Ib7, AAC(6')-IIa, AAC(6')-IIc, AAC(6')-30/AAC(6')-Ib', AAC(6')-Ie-APH(2'')-Ia	**36 (100%)**
APH	7	APH(3'')-Ib, APH(3')-Ia, APH(3')-IIIa, APH(3')-VIa, APH(3')-XVa, APH(6)-Id, aphA15	**36 (100%)**
ANT	8	ANT(2'')-Ia, ANT(3'')-IIa, ANT(3'')-IIb, ANT(3'')-IIc, ANT(3'')-II-AAC(6')-IId, ANT(6)-Ia, ANT(6)-Ib, ANT(9)-Ia	**36 (100%)**
add	21	aadA2, aadA3, aadA4, aadA5, aadA6, aadA6/aadA10, aadA7, aadA8, aadA8b, aadA10, aadA11, aadA12, aadA13, aadA15, aadA16, aadA17, aadA22, aadA24, aadA25, aadA27, aadS	**36 (100%)**

Supplementary Table S9. Diversity and prevalence of clinically relevant M-L-S families identified by CZ ID workflow in 36 wastewater metagenomes.

**Gene family**	**No. of variants**	**Variants detected**	**Positive samples (n = 36)**
erm	14	ErmA, ErmB, ErmF, ErmG, ErmT, ErmW, ErmX, erm(32), Erm(33), Erm(35), Erm(47), Erm(49), Erm(50), Erm(51)	**36 (100%)**
mph	6	mphA, mphB, mphE, mphF, mphG, mphI	**36 (100%)**
ere	4	EreA, EreA2, EreB, EreD	**36 (100%)**
lnu/lin	9	lnuA, lnuB, lnuC, lnuD, lnuF, lnuG, LnuH, LnuP, linG	**36 (100%)**
Others	1	Mrx	**35 (97.2%)**

Supplementary Table S10. Diversity and prevalence of clinically relevant fluorochinolones families identified by CZ ID workflow in 36 wastewater metagenomes.

**Gene family**	**No. of variants**	**Variants detected**	**Positive samples (n = 36)**
qnrS	5	qnrS2, qnrS15, qnrS6, qnrS7, qnrS9	**34 (94,5%)**
qnrVC	4	qnrVC1, qnrVC4, qnrVC5, qnrVC6	**28 (77.7%)**
qnrB	4	qnrB16, qnrB19, qnrB35, qnrB38	**4 (11.1%)**
qnrD	1	QnrD1	**1 (2.8%)**
